# What is intended by the term “participation” and what does it mean to people living with dementia? A conceptual overview and directions for future research

**DOI:** 10.3389/fresc.2022.952722

**Published:** 2022-08-11

**Authors:** Sarah Kate Smith, Emma Louise Wolverson, Gail Anne Mountain

**Affiliations:** ^1^ Health, Wellbeing & Lifesciences, Sheffield Hallam University, Sheffield, United Kingdom; ^2^ Health Sciences, University of Hull, Hull, United Kingdom; ^3^ Clinical Psychology for Older People, Humber Teaching NHS Foundation, Hull, United Kingdom; ^4^ Health Studies, University of Bradford, Bradford, United Kingdom

**Keywords:** dementia, participation, outcome measurement, citizenship, human rights

## Abstract

Policy continues to emphasise the importance of wellbeing in dementia. However, there is a vital need for psychosocial interventions that can promote positive outcomes to enhance “living well with dementia”. Our developing understanding of what people living with dementia report as being important to them, has resulted in new interpretations of what constitutes wellbeing including constructs such as “growth”, “purpose” and “participation”. These exciting and important constructs are not currently captured by outcome measures within dementia research. This limits our understanding of the value of psychosocial interventions. This paper explores the concept of participation and how continued participation in social life can make a difference to the rights of people living with dementia as citizens. We will firstly consider why participation is important for how we might measure outcomes in dementia research and care. Secondly, we will explore how we might measure participation. Finally, we will consider the value of participation as a psychosocial outcome in future research.

## Introduction

People with dementia report that remaining socially connected with family, friends and the wider community is key to living well and sustaining quality of life (1). Participation has the potential to uphold “personhood” (2) through promoting feelings of self-esteem and belonging. Participation in social life can result in feelings of pleasure, respect, dignity, and recognition (3). To enact ones “right” to continue to participate in society beyond a dementia diagnosis, as active and engaged citizens, remains a key national and international policy driver (4–6). A human rights-based approach to dementia is based in autonomy, empowerment, dignity, social inclusion, participation, and non-discrimination (5). This human rights movement in dementia is championed by advocacy organisations including World Health Organisation (WHO) (7), Alzheimer's Disease International (ADI) and Dementia Alliance International (DAI) (8), Alzheimer's Europe (9) and the Dementia Engagement and Empowerment Project (DEEP) (10). Closely aligned to the concept of human rights is that of “citizenship”. Citizenship while living with dementia involves the rights of individuals; to inclusivity and recognition and is continuing to gain recognition (11). Viewing dementia through a citizenship lens acknowledges the contribution that people living with dementia can bring to everyday social situations including self-advocacy, community groups and to the research community as experts by experience (12). Concepts of citizenship in dementia has shifted from viewing citizenship as a status bestowed on people, to that of a dynamic practice, enacted through individual “participation” in everyday life (13). Models of citizenship challenge researchers to capture and measure participation in society, to create and develop novel ways that enable people living with dementia to participate as equal citizens and to create interventions and outcomes that are appropriate and accessible to the populations they are intended for (14).

However, a number of barriers exist that can impact the maintenance or continuation of participation in life by those with a diagnosis of dementia. The most obvious challenge is the cognitive changes experienced in dementia which can lessen a person's ability to continue creating their own activities as well as diminish the desire to maintain “participation” in what they once enjoyed which in consequence may lead to social isolation, insecurity, apathy, and anxiety (15). A much more subtle but powerful barrier to participation are the socially constructed consequences of dementia which include stigma, marginalisation, isolation, loneliness, discrimination, and an imbalance of power which views people with dementia as “other” or “lesser” and not able to participate fully in society (16–18). People living with dementia are still frequently defined by what they can no longer achieve (19). This “malignant social psychology” (2) and the loss of personhood experienced by so many people with dementia, can reduce opportunities to participate in social life and result in withdrawal and further decline. People living with dementia consistently report that whilst their attitudes, beliefs, opinions, and preferences remain the same, it is the reactions of “others” to their diagnosis that makes them feel different (19). Additionally, and although often done with the best intentions, “others” may overtake tasks to help but this can render people living with dementia disempowered with feelings of worthlessness and underachievement. This can lead to “excess disability” (2) and result in the person with dementia experiencing apathy, depression and “learned helplessness” (19). Although, it should be acknowledged that changes in the brain are also responsible for feeling apathetic as apathy develops in the part of the brain responsible for motivation, planning and sequencing of tasks (20).

What we need are ways of supporting people with dementia to continue to participate (in common with the rest of society) and ways of enabling people with dementia to authentically record how their needs for participation are met (or not) through various interventions.

This paper will emphasise the importance of exploring ways to enable people living with dementia to continue participating in society, relationships and the things that matter to them.

The aim of this paper is to explore the term “participation” and what this means for people living with dementia and to highlight the need for a collective effort to increase understandings of how continued “participation” in social life is valued and can make a positive difference to people living with dementia. We will begin by outlining current conceptions of participation, followed by issues encountered when attempting a unified definition of “participation”. Challenges of measuring concepts like “participation” as an outcome in dementia research will then be considered. The paper concludes with recommendations for further research and developments.

### What is “participation”?

The concept of participation is increasingly used in health and social care contexts since the WHO introduced it in the International Classification of Functioning, Disability and Health (ICF) in 2001 (21). The ICF defines participation as “involvement in a life situation of people in the actual context in which they live” (22). Here we see the difference between “activity” and participation, the ICF states that “activity” describes “*the execution of a task or action by an individual”* whereas *“*participation” involves “*a person”s involvement in a life situation”*. The World Health Organisation suggests that whilst policymakers cannot create “participation”, more can be done to create “spaces” that enable and encourage social participation, especially when involving vulnerable and marginalized communities, such as older people living with dementia (23). Yet, health professionals, researchers, and society generally, have often used the concepts of “participation” and “social participation” interchangeably or with social engagement, social connectedness, social capital, social support, social network, social integration, and community involvement. Piškur et al. (22), does question whether the IC classification of “participation” actually differs conceptually to what we consider to be “social participation” or are we actually talking about the same concept? They propose that the ICF definition raises uncertainties regarding whether participation is an objective condition, thus measurable, and differs completely from social participation, which is a subjective experience, involving decision making and increasing the well-being of self and the community (22). Some argue that the concept of participation is objective thus measurable, whereas “social participation” is subjective thus difficult to capture. There is no current definition of “individual participation”, or “social participation”.

A comprehensive literature review undertaken by Brodie et al. formed part of a major UK research project called “Pathways through Participation: What creates and sustains active citizenship?” (24). This initiative was led by the National Council for Voluntary Organisations (NCVO) in partnership with the Institute for Volunteering Research (IVR) and Involve (a National Institute for Health Research initiative to support active public involvement in NHS, public health, and social care research) in an attempt to understand the different forms of participation. The findings led to identification of three main forms of participation, public, social, and individual (24). Public participation refers to the engagement of individuals within structures and institutions of democracy including political, civic and governance; social participation refers to collective activities that individuals may be involved in as part of day-to-day life including community groups and volunteering; Individual participation refers to the choices and actions that individuals make as part of their daily life that illustrate the kind of society they want to live in including buying fair-trade products and donating to charities (24).

Participation is thought to play an important role in successful ageing throughout the life course. A systematic review undertaken to conceptualise the related terms of ageing well, positive ageing, healthy ageing, effective ageing and optimal ageing, identified participation as a common construct across all studies (25). The review concludes that the concept of “participation” is not well defined in the successful ageing literature and is often used interchangeably with engagement, social support, social networks, meaningful activity, and occupation (25). Although, it should be acknowledged that the concept of “successful ageing” is often not applied to those living with dementia.

Primary research by Margot-Cattin et al. (26), describes the first step in questionnaire development to measure Participation in Activities and Places Outside Home (ACT–OUT) with 26 older adults without cognitive impairment and five older adults living with dementia. The questionnaire targeted places older adults visit; aspects influencing participation, such as transportation, familiarity, and risk perception; and questions on perceptions of self. The findings describe the decreasing abilities or opportunities to participate and the associated losses of meaning and wellbeing experienced by people living with dementia. The authors propose that participation in activities and places outside the home are underexplored and there has been a lack of systematic approaches towards collecting data on people living with dementia and their continuing participation outside the home (26). Yet, the development of the questionnaire to evaluate ACT-OUT involved interviews with 26 participants without dementia (26), raising questions regarding how the questionnaire can have validity for those with dementia.

Other researchers argue that the extent of an individual's social participation is adaptable depending upon circumstances and has the potential to impact health and wellbeing (27). It has also been suggested that access to and engagement with technology by older adults may now play an important role in participation (27). In their mixed-methods study of social participation with people with dementia and without dementia, Gaber et al. (27), report that social participation is correlated with numerous positive health outcomes, including psychosocial wellbeing and the prevention of cognitive decline. They also note that, living in an increasingly digitised society, particularly following the Covid-19 pandemic, requires engagement with technology to maintain our social participation. The findings suggest that digital technology can enhance social participation, reinforce existing social relationships, and reduce social isolation among older people living with dementia (27).

There is a wealth of literature focused on the concept of participation (22, 24, 25) in the general population, but limited evidence that includes populations living with dementia (26, 27). Nevertheless, the conclusion that social participation is subjective and affected by environmental, social, and personal factors, is important as it suggests that definitions may not be generalisable across populations (22). Yet, the limited inclusion of dementia from the existing literature on participation highlights the urgency of reaching a satisfactory definition of the term. Environmental, social, and personal factors are all relevant to people living with dementia but there remains no useful way to authentically capture what participation means in psychosocial dementia research.

### What does “participation” mean to people living with dementia?

Despite gaining better understandings of what “participation” means to people living with dementia, we are lacking research evidence to confirm this, participation has not been defined for people with dementia, and there remains no effective ways of capturing participation for this group. Consultations have taken place to find out what people living with dementia consider to be meaningful outcomes for them when participating in psychosocial and clinical dementia research (28). Consultations involving twenty-five people with dementia from nine European countries found that people living with dementia wish to participate in interventions that enhance their well-being, confidence, health, social participation, and human rights (28). This Pan-European consultation involving discussion with people living with dementia has increased our understanding about meaningful outcome measures in dementia research suggesting new instruments are needed to capture positive outcomes (28).

Reilly et al. used the Delphi approach with 21 people living with dementia, 58 care partners, 137 relevant health and social care professionals, 60 researchers and 12 policy makers to reach consensus on a set of core outcomes for dementia research (29). The results identified 54 outcomes that were grouped into the following categories: Self-Managing Dementia Symptoms; Quality of Life; Friendly Neighbourhood & Home and Independence. The number of items under each of these categories were reduced to a final 13 outcomes considered to be important to participants. Although the concept of participation was not acknowledged through this consensus, we propose that it could meaningfully overlap with the identified concepts of “importance of relationships, communication, meaningful activities, and a sense of who you are”. Could this mean that “participation” is not important to people living with dementia despite the evidence saying otherwise or does this mean that other terms are used by the general public when talking about participation, and we are therefore discussing the same concept in different terms? It may be that the values put forward by people living with dementia were diluted given that the Delphi exercise also involved 58 care partners, 137 relevant health and social care professionals, 60 researchers, 12 policy makers. Although, the research team clarify that the perspectives of people living with dementia were included throughout the research programme, often as co-researchers (30). This research offers a good example of involving people living with dementia in research and rigorous consensus reaching regarding the core outcomes that are valued, although the project interest is firmly focused on the community. The Reilly et al. research (29) forms part of the COMET programme of work (31) which aims to facilitate the development and application of “core outcome sets” (COS). Rigorous methods that measure core outcome items can only enhance comparisons for effectiveness, making trial evidence more useful (29) but also highlight the gaps that remain when attempting to meaningfully capture positive outcomes in psychosocial dementia research. Many of the existing measures at the disposal of psychosocial dementia researchers have been psychometrically tested and validated when assessing what is important to people living with dementia yet they have been developed based on the majority assumptions of health professionals, researchers, and care partners.

### What outcomes are being measured in dementia research and are we measuring what the intervention is intending?

There is limited consensus in psychosocial dementia research regarding what domains are important to measure (29) and a reliance on well cited validated measures is maintained in the absence of appropriate alternatives. A recent systematic review intended to identify a range of outcomes that are relevant to people with dementia, care givers and health professionals, identifying 32 outcomes across seven domains considered to be important (32). The findings indicate that the most frequently utilised outcomes reflect memory decline and cognition, practical challenges such as accessing ADL information or the ability to complete ADLs alongside personal aspects such as maintenance of a persons' autonomy, identity, and QoL (32). Activities of Daily Living (ADL’s) are different to the ability to continue participating in society. The measures we have to capture ADL’s are multiple, but as more people living with dementia are getting involved in research and we are hearing their voices, they are saying that their ability to continue to participate is important to them, yet we have no authentic means of capturing participation for this group of people. The concept of “participation” was not identified in any of the reviewed studies although “social engagement” was an identified concept in three of the reviewed papers and identified as important by two people living with dementia, one caregiver but no health professionals (32).

In a review of outcome measures used in studies involving people living with dementia, questions were raised regarding the relevance of the domains being measured and whether they were capturing aspects of the experience that were important to people living with dementia (33). The review concluded that the range of measures is highly heterogenous, and doubt remains regarding which domains are most important that little agreement could be reached about which measures would be most useful. Further, in a review of disease-modifying interventions for people with dementia, it was reported that out of 125 identified trials, 81 different outcome measures were used (34). The researchers also spoke with people with dementia and their caregivers to identify what measures would be important to them. They concluded that individual differences and personality traits have an impact on the way a person responds, and therefore finding a way to capture this is important (34).

Another scoping review of outcome measures used in RCT's of non-pharmacological interventions with people living with dementia highlight an inconsistency in the use of outcome measures (35). Cognition continues to be prioritised over other domains irrespective of whether improved cognition is the intended focus of the intervention and despite previous research highlighting the potential of interventions to improve aspects of quality of life above allaying disease progression (35).

A large, well designed RCT of Reminiscence Groups found beneficial effects for people with dementia such as autobiographical memory, relationship quality and quality of life but these effects were offset by raised anxiety and stress in their carers (36). These results were unexpected and were in contrast to the reported enjoyment and benefits reported by participants and facilitators during the group the sessions. This could indicate that the measures being utilised were not representative of the intended benefits of the intervention as there were no tools available to measure “enjoyment”, “engagement” or “participation”. Additionally, it was noted through this RCT that there is an argument for interventions involving people with dementia to be evaluated for their immediate, within-session effects, rather than focusing on longer-term changes, pre-post and follow-up (36).

In order to comprehensively evaluate the impact of any intervention, the outcome measures should reflect the lived experience of the condition (37) and in dementia research this should not be limited to cognitive, functional and QoL measures. In their evaluation of outcome measures from a decade of dementia diagnosis and treatment research trials, Harrison et al. found substantial heterogeneity in assessment, poor descriptions of assessment tools and a continued reliance on cognitive measures (37). This finding is supported by the review by Couch et al., that included Mild Cognitive Impairment (MCI) as well as dementia, found the highly heterogeneous characteristics of participants recruited to the types of interventions being tested and the inconsistent use of outcome measures make it difficult to establish the effectiveness of one intervention over another as no useful comparisons can be made (35). To ensure a robust evidence base is developed, further research is needed to better understand if existing instruments are appropriate and which instruments should be prioritised over others (33, 35).

A European consensus has been reached regarding the most appropriate existing outcome measures for psychosocial intervention research (38, 39), although this is currently being updated. However, the non-cognitive domains being assessed are more general and include Activities of Daily Living (ADL's), Mood, Behaviour, Quality of Life (QOL) and Carer Burden (40), and researchers continue to measure these domains as there remains an expectation to do so which can be promoted by funders or regulatory authorities.

### How do we know if we are measuring participation in dementia research?

Whilst people living with dementia have highlighted participation as being a meaningful outcome for them the concept remains ill-defined and there are no tools to measure it. Psychosocial dementia research can aim to capture insights before measures can be constructed although research still prioritises cognition and the improvement over time as the main aim. Many of the studies that have taken place may actually be measuring participation through participants ability to independently undertake Activities of Daily Living (ADL), but ADLs are activities that need to be maintained to remain independent and should not be labelled as anything else. Nevertheless, ADL and QoL measures in particular, are utilised in an attempt to capture fluctuations in participation but questions remain regarding overlapping meanings and how concepts are defined and by whom.

It has been noted that many of the tools available to psychosocial dementia researchers measure neuropsychiatric symptoms including depression, anxiety and agitation thus “inferring” wellbeing by the absence of these particular symptoms (41). This is problematic for a number of reasons. First, many of the measures available to psychosocial researchers were developed for non-psychosocial and clinical trial research (42) and not developed to measure psychosocial outcomes in dementia. Second, these measures are not intended to measure the positive concepts of wellbeing that psychosocial dementia researchers are trying to capture as many are generic measures rather than dementia specific. Consequently, and in the absence of tools that capture positive concepts, researchers have had to employ measures that are not specific to a dementia population. Third, measure development has not adequately ensured that the views of people living with dementia are included nor do they include a focus on lived experience that research consistently demonstrates is of the utmost importance when people living with dementia are asked about their lives (43).

It is clear that the concept of “participation” is subjective, ill-defined, and challenging to capture as psychosocial dementia researchers lack appropriate tools. There is no widely accepted measure of “participation” despite the evidence suggesting that this is important to people. Because there is no common definition of “participation” there are no methods to assess participation or its constituent parts. Much of the evidence base is impeded as existing instruments to capture wellbeing, life satisfaction and quality of life, although sharing some similar constructs, the terms tend to be conflated thus will ultimately yield varying results. This lack of consistency across studies is methodologically limiting, rendering it challenging to draw conclusions regarding the constructs of living well with dementia, and to identify what works and for WHO (44).

### “In the moment” participation: a call to action for potential ways forward

People living with dementia have spoken about living in the “here and now” and the importance of being “in the moment” (45). A person may participate in an activity or social event and in that moment derive significant enjoyment and pleasure from their participation. However, later they may no longer remember some or even all of that experience. A person's experience of their condition can and will fluctuate rendering reflection and self-report of the past month or even week challenging and inaccurate and aside from observational tools, measures rely upon on recall. This has been a significant challenge in dementia research, due to the dynamic and complex nature of participation as well as psychosocial interventions, where static, retrospective measures are failing to capture the rich and valuable experience data to its full effect (46). Existing assessment tools have been used to try and capture “in the moment” participation in activity with people living with dementia. Some examples include Global Positioning Systems (GPS) to assess outdoor mobility, travel patterns (47, 48), sensors, video cameras or smart home technologies may be used to assess the performance of Instrumental Activities of Daily Living (IADL) (49) apathy (50), movement patterns (51) and sleep-wake activities (52, 53). These tools offer some ways to capture participation, as long as they are easy to use or unobtrusive and acceptable to people living with dementia (54, 55). Currently GPS systems measure activity, not participation, and should not be mistaken for this, nonobtrusive or not. However, future work is required to identify how technologies can be readily used in acceptable and non-obstrusive ways and can be adapted to capture “in the moment” particiption.

Although it is clear that people living with dementia can give accounts of their lived experiences and should never be excluded from developments that are aimed to enable them to live well, there remains no appropriate ways to meaningfully capture these accounts. Psychosocial dementia researchers continue to be hampered by traditional qualitative methods such as questionnaires, semi structured interviews and focus groups which rely upon the coherent articulation of users' experiences and views of health. The skills required for communicating through writing, reading, and speaking can be compromised in people living with dementia. In addition, many participants living with dementia are asked to reflect on recent events of the past week or month which is an unacceptable request and assumption continually made by researchers, as highlighted throughout this paper.

Designing and developing ways to capture, define and assess the lived experience of participation requires methods that are sensitive and flexible to change. Existing measures do not consider the person with dementia's fluctuating experience at different points in time nor that individual differences and personality traits have an impact on responses as they were not developed nor intended for a dementia population, thus finding ways to capture this would be important (34). There is also a plethora of existing evidence that indicates the appropriateness of digital technology when researching with people living with dementia (28, 56–58) and the potential for capturing “in the moment” experiences of participation provide exciting future opportunities. Ultimately, ways forward can be drawn from other collaborative actions that have reached a European consensus on the operationalisation of concepts and directions for future research and practice (59).

## Conclusion

The aim of this paper was to discuss and debate existing thinking around the concept of “participation” and what this means for people with dementia. It is evident that there is no unified definition of participation despite people living with dementia reporting that the concept is important to them. How to capture the lived experiences of people living with dementia is most often compromised as existing measures often require the person to reflect on past events which is not always achievable or appropriate. Furthermore, a mismatch between the goals of contemporary psychosocial dementia research and the available tools to assess benefit has also been highlighted. If dementia researchers had appropriate tools to measure benefit (or not) the impact of psychosocial dementia research interventions would be far more explicit rather than inferred through increased participation as a result of an intervention. This paper adds to the debate regarding what is important to the lives of people living with dementia and how these perspectives are reflected in the development of new methods of assessment.

## Data Availability

The original contributions presented in the study are included in the article/Supplementary Material, further inquiries can be directed to the corresponding author.
